# A Review of the Mechanisms of Ventricular Arrhythmia in Brugada Syndrome

**Published:** 2010-09-05

**Authors:** J Bhar-Amato, LM Nunn, PD Lambiase

**Affiliations:** The Heart Hospital, University College Hospital and Institute of Cardiovascular Sciences, UCL, 16-18 Westmoreland Street, London W1G 8PH

**Keywords:** Brugada syndrome, ventricular arrhythmia, right ventricular outflow tract, depolarisation, repolarisation, transmural gradient, sodium channel

## Abstract

Brugada syndrome (BrS) is characterised by the triad of coved ST elevation, lethal ventricular arrhythmia in an apparently structurally normal heart. The precise mechanisms responsible for the coved ST elevation and ventricular arrhythmias in this disease have been debated since its initial description in 1992. Indeed the recent recognition of early repolarisation J wave disorders linked to primary VF broadens the mechanistic importance of BrS in sudden cardiac death. It may lie on a spectrum of early repolarisation pathology which is becoming increasingly recognised as a marker of premature cardiovascular death. Mechanistically, abnormalities of both depolarisation and repolarisation in the right ventricular outflow tract, and heterogeneities of conduction between the endocardium and epicardium have been implicated in the electrographic manifestations of BrS and arrhythmogenesis.

The initial belief of BrS as a single autosomal dominant ion channel disorder has been challenged. It has become apparent that sodium channel mutations only account for a maximum of 30% of cases and structural myocardial abnormalities have now been described in what was previously thought to be a purely functional condition. It is highly probable that BrS is an umbrella diagnosis for a number of conduction and repolarisation abnormalities which manifest as this syndrome and the coved ST elevation represents the final common pathway of both ion channel and structural derangements. This review will discuss the issues surrounding the mechanisms of lethal arrhythmia in BrS and summarise both basic science and clinical research findings.

## Introduction

The Brugada syndrome (BrS) was formally characterised as a distinct clinical entity in 1992 [1] and is thought to be responsible for 20% of sudden death in people under 50 years of age with apparently structurally normal hearts [2]. Inherited in an autosomal dominant fashion with incomplete penetrance, symptoms tend to manifest at an average age of 40 years in the form of syncope, nocturnal agonal respiration, ventricular arrhythmia and sudden death [3]. It has a significant male preponderance but its true prevalence (estimated at 1-5 in 10,000 worldwide [4] is difficult to quantify not only because of its transient signature electrocardiographic signs but the failure to correctly identify or report cases of suspected sudden arrhythmic death and the lack of subsequent family screening. Ventricular arrhythmia in the syndrome generally takes the form of polymorphic ventricular tachycardia (VT) or ventricular fibrillation (VF) with the right ventricular outflow tract (RVOT) identified as the principal arrhythmogenic site.

Abnormalities of repolarisation or depolarisation and heterogeneities in conduction both within the epicardium and between the epicardium and endocardium have been proposed to account for the electrocardiographic abnormalities and ventricular arrhythmia. There is increasing evidence that each of these mechanisms may play a role. BrS as a purely functional ion channel disorder is also being questioned by contradictory evidence gathered from cardiac biopsies, autopsy specimens and new high resolution imaging modalities.

A re-entrant mechanism perpetuates ventricular tachycardia in BS, and requires an initiating mechanism or 'trigger' and a substrate capable of maintaining it. Since the right ventricular outflow tract is the main patho-physiological arena in this disease, its structure and embryological orgins will be reviewed before discussing the main hypotheses of arrhythmogenesis in BrS.

## The Right Ventricular Outflow Tract

In Brugada syndrome, the classic ECG findings are present in the right praecordial leads and ventricular arhythmias are more readily induced from the right ventricle [5-8]. Moreover, signal averaged ECG data, body surface mapping, endocardial and epicardal recordings in the conus branch over the RVOT have identified conduction delay in this area [5,9-12]. Extrasystoles triggering arrhythmia have also been mapped and ablated in this region  [9].

The right and left ventricles and RVOT are formed along the craniocaudal axis of the heart along which signalling and other critical regulatory processes are polarised. This is suggested to account for anatomical differences and electrophysiological heterogeneity in the adult [13].

A level of electrophysiological heterogeneity in the heart is vital for optimal cardiac function, facilitating co-ordinated contraction and relaxation. This heterogeneity is rooted in embryological development and modulated gene expression resulting in regional differences in tissue structure and ion channel distribution. Structural and electrophysiological heterogeneities exist on multiple levels: (i) between apex and base, (ii) left and right ventricles, (iii) RVOT and right ventriclular (RV) body, and (iv) between epicardium and endocardium [13]. Critical thresholds in these regional electrophysiological differences exist, which if crossed, lead to excessive conduction delays, unidirectional block and re-entry. The stability of the resultant re-entant circuit is itself dependent upon regional action potential duration (APD) restitution properties.

The adult RVOT itself develops from the embryological outflow tract, which is formed at a later stage from the rest of the heart, and arises from the second heart field, an origin distinct from the ventricles (formed from the first heart field), with added influence from migratory cardiac neural crest cells. These developmental differences may account for the RVOT being critical in BrS pathogenesis. The program regulating cardiac neural crest development and formation is controlled by multiple distinct signals that originate in the pharyngeal endoderm and local mesoderm and the neural crest cell death program also plays an active role in stimulating outflow tract myocardialization [14-15]. Likewise, Connexin 43 (Cx43) is involved in the differentiation of some cardiac crest cell lineages; among them, gap junctional communication has been linked with the differentiation of cardiac myocytes [16]. Differences in Cx43 expression exist between the epicardium and mid/endocardium which could lead to transmural conduction discontinuites if neural crest migration is disrupted [17]. Significant regional and/or transmural differences that arise from such disruptions may contribute mechanistically to the principle theories of arrhythmogenesis in BrS: the repolarisation and depolarisation hypotheses.

## The Repolarisation Hypothesis

**Figure 1:**
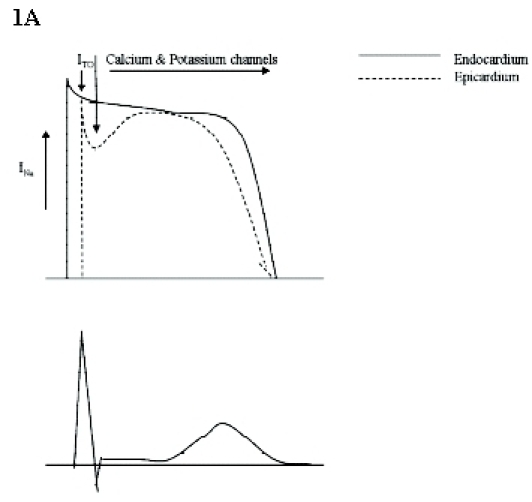

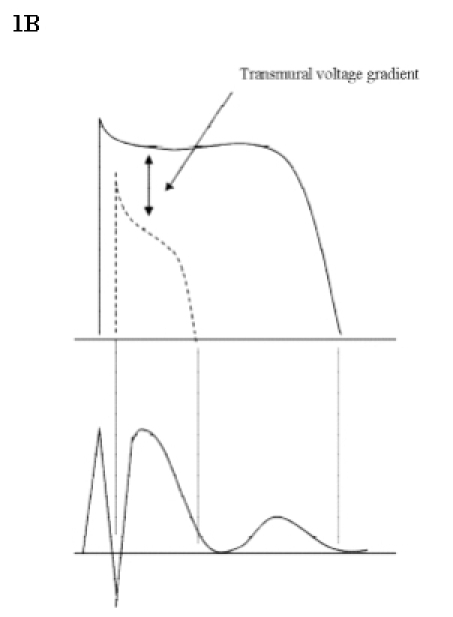

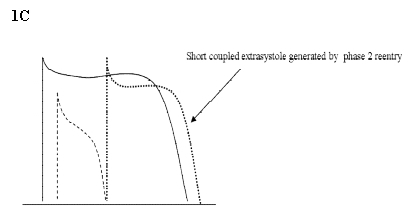

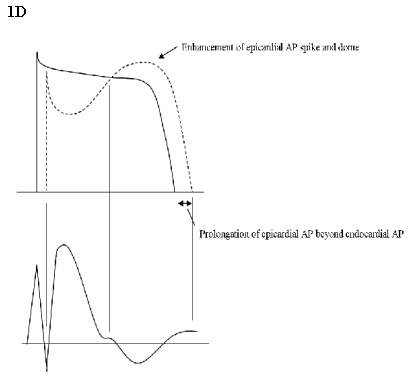
The repolarisation hypothesis: Enhanced I_TO_ expression in epicardium compared with endocardium results in a spike and dome AP morphology in the epicardium under normal circumstances (A). With reduction in sodium current, the amplitude of the phase 0 upstroke is reduced. Due to enhanced epicardial I_TO_ expression, the phase 1 downstroke reduces the opening probability of calcium channels responsible for the early phase 2 plateau. This results in loss of the AP dome (B). This creates a transmural gradient between the endocardium and epicardium. This is thought to cause saddleback or coved ST elevation with the T wave remaining positive. Loss of the dome in some areas of epicardium and preservation in others facilitates phase 2 re-entry resulting in a closely coupled extrasystole at the site of epicardial AP dome abbreviation (C). Fig 1D shows the enhanced epicardial AP spike and dome with prolongation beyond the endocardial AP, demonstrating how this can have a corresponding surface ECG representation of both coved ST elevation and T wave inversion.

This hypothesis has received its main support from experimental models and posits that normally, in contrast to endocardium, the epicardial action potential (AP) has a prominent phase 1 resulting in a spike and dome morphology [18] (Figure 1A). This is thought to be the result of a more pronounced expression in epicardial cells of the transient outward potassium channel (I_TO_), largely responsible for the rapid downstroke of phase 1. If there is a reduction in the amplitude of phase 0, this will result in a lower voltage level at which phase 1 begins. This affects the activation/inactivation kinetics of both I_TO_ and the availability of the L-type calcium channel involved in phase 2, resulting in a loss of the AP dome and shortening of the epicardial AP duration (APD). The less pronounced phase 1 downstroke of endocardium due to lower I_TO_ expression allows for conservation of the AP pattern, even with some reduction in phase 0 amplitude (Figure 1B). The transmural gradient during phase 1 between the epicardium and endocardium was shown in the canine wedge experiments by Antzelevitch and Yan, to contribute to both J point and ST elevation on the surface ECG [18].

The loss of the epicardial AP dome in some areas and retention in others results in the 'trigger' mechanism: a closely coupled extrasystole generated by phase 2 re-entry (Figure 1C). The increased dispersion of repolarisation between endocardium and epicardium and within the epicardium provides a further substrate for maintenance of arrhythmia.

Although this heterogeneity in AP duration could account for the corresponding ST changes in Brugada syndrome, it does not explain the T wave inversion that accompanies type 1 (right praecordial coved ST elevation) and sometimes type 2 (right praecordial saddleback ST elevation) electrocardiograms. It has been proposed that both an exaggeration of the notch and a prolongation of the epicardial AP beyond the endocardial action potential would be necessary to observe the raised J point, ST elevation and inverted T wave [18,19] (Figure 1D).

### In Vivo Human Studies

Although most of the data supportive of the repolarisation hypothesis has been derived from pharmacological manipulation of the canine wedge preparation, interesting observations have been made in man. Nagase et al reported a patient in whom they had inserted an epicardial electrode in the great cardiac vein a small distance from an endocardial catheter [11]. During augmented ST elevation, the epicardial but not endocardial activation-recovery interval (ARI - a surrogate of APD) shortened. Kurita et al demonstrated abnormal spike and dome configurations of RVOT epicardial monophasic action potentials (MAPs) in the RVOT of 3 Brugada patients (all with type 2 ECGs), noting incomplete depolarisation in phases 0 and 1 and a deep notch in phase 2 [20]. This was not found endocardially or in control patients. They could not, however, demonstrate any loss of the AP dome and shortening of the epicardial action potential and instead found a MAP and repolarisation of longer duration in the epicardium compared with endocardium due to delayed dome formation. Although this could account for the reversal of the gradient in phase 3 necessary for the typical inverted T-wave, it raises doubt over the repolarisation theory. Abnormal MAPs were recorded in neighbouring epicardial sites over about a 2 cm radius of the RVOT but not in other sites in the RV anterior wall and LV anterior and lateral wall. Although a novel in vivo demonstration of alteration of MAPs in BrS, the recordings made from corresponding epicardial and endocardial sites were not done simultaneously, with the epicardial MAPs being collected during open chest surgery.

In support of I_TO_ being a key channel involved in the Brugada ECG pattern, the right ventricle has a higher density of this channel compared with the left ventricle and it is suggested that the thinner endocardium of the right ventricle relative to its epicardium results in a more marked spike and dome pattern in this region [21]. The marked prevalence of the syndrome in males compared with females may also be explained by an overall increased expression of I_TO_ in men [22]. The administration of quinidine, an effective I_TO_ blocker, can lead to normalisation of the ECG and has suppressed arrhythmic activity in experimental models and human patients [23-26]. The normalisation of the ECG at increased heart rates may be due to the slow reactivation kinetics of I_TO_ rendering it inactive at lower diastolic intervals leading to restoration of the epicardial action potential dome. However, exaggeration of the Brugada ECG changes at higher heart rates has also been observed.

An outward shift in the balance of currents in the early phase of the action potential could account for the abbreviated epicardial action potential. Thus theoretically, a reduction in INa or the L-type calcium channel, an increase of I_TO_ and/or any time dependent potassium current could result in the observed ECG pattern. For instance, sodium channel blockers can reveal the Brugada ECG and acetylcholine, which suppresses ICa and increases potassium current, can augment these changes. SCN5A, the gene encoding for the a subunit of the Nav1.5 voltage gated sodium channel and in which a loss of function mutation is seen in about 20% of Brugada patients, is more abundantly expressed in endocardium compared with epicardium [27]. This results in a lower upstroke velocity in the epicardial action potential which, together with increased I_TO_ expression, generates normal transmural heterogeneity. Reduced sodium current with preserved outward potassium current would be expected to increase the transmural gradient.

### Mode of Onset of Ventricular Arrhythmia

The phenomenon of phase 2 re-entry triggering arrhythmic activity in the human right ventricle in vivo was first demonstrated by Thomsen et al. 83% of their study patients (not affected by Brugada syndrome) showed J-point elevation, ST elevation and T-wave changes in the sinus beat preceding ventricular extrasystoles and non-sustained ventricular tachycardia [28]. The mean coupling intervals of these extrasystoles was 300 to 505 msecs rather than the short coupling intervals expected of phase 2 re-entrant beats. It was argued that this may be due to the initial phase 2 re-entrant beat being concealed in the T wave of the preceding sinus beat, with the coupling interval of the following extrasystole being measured [29].

The mode of onset of ventricular fibrillation in Brugada patients with ICDs was studied by Kakashita et al. 33 episodes of spontaneous VF in seven patients were analysed [30]. Before VF onset, frequent ventricular extrasystoles were observed in the majority of episodes. These extrasysloes were almost identical to the initiating beat of VF. In the remaining episodes, preceding extrasystoles were not seen but the initiating beat of VF was morphologically similar to extrasystoles observed on remote occasions. Different VF episodes in the same patient were initiated by almost identical extrasystoles, and in two patients, initiating extrasystoles were morphologically similar, all suggesting a common ventricular focus. Coupling intervals were on average 388 ms, close to the end of the T wave, rather than the short intervals expected of those resulting from phase 2 re-entry. Kasanuki et al also documented >300 msec coupling intervals but also reported an increase in ST elevation in right praecordial leads prior to VF onset [31], suggesting that the ST changes and extrasystoles are linked by a common mechanism.

In the same year that Thomsen et al published their findings, however, Coronel et al presented an experimental data on the explanted heart of a 34 year-old male Brugada patient, who had undergone transplantation due to a high incidence of VF [19]. Using epicardial strips and non-contact endocardial mapping, they failed to demonstrate a transmural repolarisation gradient and instead showed conduction delay in the right ventricular outflow tract, with the first beat of induced VF having a subendocardial, rather than subepicardial origin. Although ST elevation was not present during mapping, computer simulations demonstrated that RV conduction delay alone in this case could result in a typical Brugada ECG, without there being alteration in action potential duration, lending support to the other main theory in BrS, the depolarisation hypothesis.

### The Depolarisation Hypothesis

**Figure 2:**
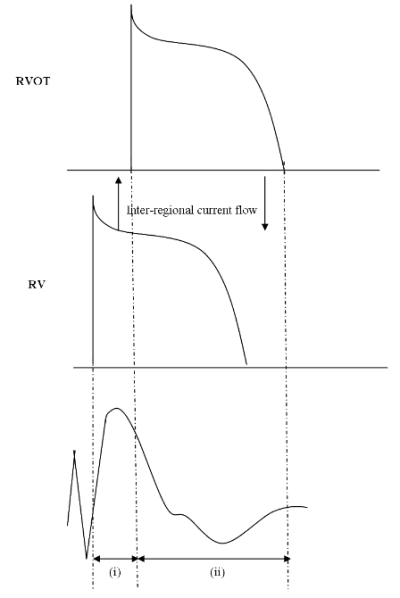
The depolarisation hypothesis: Activation is delayed in the RVOT. Prior to the onset of the AP in the RVOT, the RV, having depolarised, acts as a source of current (i). This current travels to the RVOT, acting as the sink, inscribing a positive deflection on the ECG (in the right praecordial leads).Once the RVOT depolarises and the RV is repolarising, the RVOT now acts as the source of current with the RV acting as the sink (ii). This reversal in the direction of current flow inscribes a negative deflection on the ECG (negative Twave).

This theory proposes that conduction delay in the RVOT results in unidirectional block leading to re-entry. If the RVOT AP is delayed in relation to that of the RV body, a voltage gradient develops between the two sites with the more positive membrane potential in the RV body (source) driving intercellular current to the RVOT (sink). The circuit is completed by extracellular current from the RVOT to the RV, inscribing a positive deflection on the ECG (J point and ST elevation). This source-sink mismatch is then reversed with the delayed upstroke of the RVOT AP and as the RV repolarises, the more positive gradient in the RVOT drives the intercellular current to the RV, with the extracellular current now travelling from the RV to the RVOT, inscribing a negative deflection on the ECG (T-wave inversion) (Figure 2). The delayed conduction can lead to regions of functional block facilitating re-entry.

The prolongation of the PR interval and QRS duration in a significant proportion of Brugada patients has lent some support to the concept of conduction disturbances [33]. There has also been compelling evidence from signal averaged ECG, body surface mapping, tissue doppler echocardiography and invasive electrophysiological studies.

Late potentials, representing delayed ventricular depolarisation, are prevalent in Brugada patients and have been found to coincide with spontaneous and pharmacologically induced ST elevation. Eckardt et al performed signal averaged ECGs, body surface mapping and programmed ventricular stimulation studies on sixteen Brugada patients with type 1 ECG changes at baseline, who had suffered syncope or aborted sudden cardiac death [10]. A larger area of ST elevation was found in Brugada patients compared with control patients, which increased after ajmaline administration. Late potentials were found in the majority. These two findings were correlated to the ease of VT inducibility in these patients. Takami et al found an increased incidence of late potentials among patients with coved ST elevation, a group generally accepted to be at higher risk, compared with saddleback ST elevation [12]. Antzelevitch has pointed out, however, that although late potentials are generally thought to reflect delayed depolarisation, they could also actually reflect either the exaggerated second upstroke of the epicardial action potential after the marked downstroke due to I_TO_ repolarising current in phase 1 or the concealed phase 2 re-entry generated extrasystole(s) which has failed to trigger arrhythmia [29].

Nagase et al also found delayed potentials on the epicardial surface of the RVOT, using a wire inserted into the conus branch of the right coronary artery [5]. These coincided with later potentials recorded on signal averaged ECG. The same group then studied the ARIs and repolarisation times (RT) in the RVOT endocardium and epicardium simultaneously during ventricular stimulation studies using the conus branch epicardial recordings. Ten of the nineteen patients in this latter study had a type 1 ECG at baseline and nine developed type 1 changes following pilsicainide administration. They found longer ARIs and RTs in the epicardium compared with endocardium in Brugada subjects with a type 1 ECG at baseline. Shorter epicardial ARIs and RTs compared with endocardial repolarisation times were found in the control subjects as expected, and in Brugada patients with normal ECGs at baseline. In the latter group of patients, the epicardial ARIs and RTs were preferentially prolonged after pilsicainide, becoming longer than the endocardial ARIs and RTs, which remained the same. They were unable to demonstrate an accentuation of the epicardial AP notch, loss of the dome nor any shortening of the epicardial action potential but postulated that the latter could be because not all of the RVOT was studied and there may have been shortening in some areas and prolongation in others. Significantly longer activation times were also seen in Brugada patients with baseline type 1 ECGs compared with those who had normal ECGs.

Tukkie et al hypothesised that activation delay in a particular area would cause delayed contraction which would be detected by tissue Doppler echocardiography [33]. They studied contraction timing and contractile force in sixteen Brugada patients at baseline and post flecainide administration. All these patients had normal 2D echocardiograms. Delayed RV contraction occurred and corresponded to ST elevation in patients with type 1 changes at baseline, and those who developed these changes with flecainide. A marked impairment of tissue systolic velocities, reflecting contractile force, was not detected in these patients, suggesting that selective action potential shortening was not a factor here. However, reduced ejection times were also observed in the RV compared with the LV. This is not seen in patients with right bundle branch block where there is known activation delay in the right ventricle so may imply some level of action potential shortening in the RV of the Brugada patients.

Electrophysiological testing in humans has also identified conduction abnormalities such as prolongation of the His-ventricular (HV) interval and Corrado et al demonstrated that the latest endocardial ventricular electrogram was from the RVOT [36]. Kanda et al found that patients with readily inducible VF had a longer QRS duration, a higher incidence of late potentials, longer HV intervals and a longer conduction time from the RVOT to the left ventricle compared with non-inducible group [32]. Most of the arrhythmias were induced by extrastimuli applied to the RVOT and the degree of depolarisation abnormalities was thus linked to the risk of ventricular arrhythmia. However, a number of recent population based studies have shown that VT/VF inducibility at EP study does not necessarily establish risk for arrhythmic events in Brugada patients, indicating that more sophisticated strategies are necessary to identify high risk substrates in this disease.

Postema et al performed RV endocardial mapping studies in type 1 and 2 Brugada patients [34]. They demonstrated conduction slowing and abnormal conduction velocity restitution in the right ventricle (with no significant regional differences) and showed that repolarisation characteristics were similar in Brugada patients and controls. In addition, they found not only broader but also more fractionated electrograms in the RV, citing discontinuous conduction, possibly due to subtle structural abnormalities leading to reduced intercellular coupling, as a pathophysiological aspect of the disease.

In a recent non-invasive study, it was highlighted that these depolarisation abnormalities could lead to repolarisation abnormalities [35]. At baseline, the Brugada patients they studied already had longer P, PQ and QRS intervals than controls, and vectorcardiogram and body surface mapping data found more conduction slowing in this group on ajmaline administration, during which the type 1 ECG was elicited. No repolarisation abnormalities were found at baseline: QT, QTC and Tpeak-Tend intervals were similar between Brugada patients and controls, and the conduction slowing was accompanied by JT interval shortening, representing a shortening of the duration of repolarisation. They proposed that the abnormal repolarisation resulting in an inverted Twave follows on from abnormal depolarisation.

A high resolution non-contact endocardial mapping technique was utilised in a recent study by our group and demonstrated significant regional conduction delays, a reduction in the activation gradient and the formation of lines of functional conduction block in the anterolateral free wall of the RVOT compared with the RV body and apex of Brugada patients [40]. Fractionated electrograms, suggesting tissue discontinuities, were also found in this area. Wavefront fragmentation along these areas of functional conduction block, were shown in isochronal maps to initiate polymorphic VT and subsequent degeneration into VF in 5 patients. There were also steep restitution gradients in the RV reflecting repolarisation abnormalities in the endocardium that would contribute to the arrhythmic substrate. Epicardial parameters were not measured in this study. These conduction delays could account for the fragmented QRS identified on filtered ECGs by Zipes' group which has been correlated with an increased risk of clinical VF events [37]. These studies further support the notion that a level of structural derangement may be a facet of BrS, and the potentially complex relationship between tissue and ion channel abnormalities is open to debate.

## The Role of Ion Channel Mutations and Structural Derangements

Despite a recognised autosomal dominant pattern of inheritance, mutations predominantly in sodium channel genes have only been described in 30% of the Brugada population [38]. The heterogeneous genetics of BrS may give rise to the clinical spectrum of the phenotype; with 6 genes linked to the condition at the time of writing. These include loss-of-function mutations in the cardiac sodium channel gene, SCN5A [39], and the GPD1-L gene [40], encoding for the glycerol-3-phosphate-dehydrogenase 1-like protein, dramatically altering phase 0 of the cardiac action potential. An overlap syndrome due to loss-of-function mutations in genes encoding the cardiac L type calcium channel (CaCNCA1C and CACNB2) resulting in a Brugada syndrome phenotype combined with shorter-than-normal QT intervals have been reported [41]. Furthermore, a mutation in KCNE3 encoding I_TO_, the early transient outward potassium channel current, has recently been described [42]. This leaves open the question regarding other pathophysiological processes.

Although a structurally normal heart is still required as a diagnostic criterion-there is emerging evidence that this not the case. A number of studies have demonstrated fibrosis in the Brugada right ventricle suggesting a degenerative process which may be genetic, inflammatory or infective in origin. This fibrosis may be due to premature ageing of myocytes arising from abnormal ion channel kinetics as described in the SCN5A heterozygotic mouse [43,44]. It could explain the recent MRI data suggesting RVOT dilatation in BrS cases [45,46]. Such a fibrotic process will promote conduction delays in the RV and could explain the coved ST elevation of the Brugada ECG independent of specific ion channel mutations promoting transmural repolarisation gradients. As described above, invasive mapping studies of the Brugada RV have confirmed significant conduction delays and computer simulations incorporating these delays can reproduce the characteristic surface ECG features [19]. These conduction abnormalities could also increase the arrhythmogenicity of the substrate through promoting conduction block and re-entry or causing destabilisation of VT into VF.

This identification of conduction delay in BrS raises a fascinating question, as to whether Na channel mutations which promote conduction slowing unmask the Brugada phenotype in a similar manner to a pharmacological Na channel blocker. Coronel's group points out that the identified Na channel mutations may simply be a co-factor in manifesting the disease but not the primary cause [47]. In order to identify Na channel mutations as the primary cause, one would have to show that SCN5A mutations are more prevalent in cases with less structural derangement, but this data does not yet exist. This concept relates to the 'multiple hit hypothesis' of pathophysiology where a number of factors may combine to create the disease substrate that manifests as a Brugada ECG. This has been demonstrated in long QT syndrome where a single potassium channel mutation and non-pathological ion channel polymorphism combine to create a malignant long QT phenotype but these independently, only result in minor ECG changes [48]. Due to the "physiological" reserve and compensatory ion channel currents, a single mutation or pathological change may remain subclinical until another factor/perturbation tips the balance into a pathological manifestation.

In the study by Postema et al described above, where increased electrogram duration, fractionation and activation delays were found in type 1 and 2 Brugada patients compared with controls: these findings became more marked with ajmaline in type 2 patients and conduction velocity (CV) restitution was found to be abnormal in type 1 patients, specifically with transverse, as opposed to longitudinal, propagation along myocardial fibres [34]. The abnormalities occurred with premature RV stimulation and not in sinus rhythm, not only confirming that the specialised conduction system is not involved in conduction delay in BrS, but that gross abnormalities in fibre orientation were not present in the BrS patients. This suggests that an increased intercellular coupling resistance (possibly due to tissue discontinuities like interstitial fibrosis affecting transverse impulse propagation more markedly) and/or reduced sodium current (possibly due to reduced sodium channel expression at the long axis of the myocardial fibre) could be responsible for the conduction delay. Interestingly, the presence or absence of an SCN5A mutation was not related to electrogram duration or degree of fractionation.

How structural abnormalities could mechanistically contribute to the signature ECG and arrhythmia in Brugada was demonstrated by Hoogendijk et al, who analysed the activation and repolarisation characteristics of the explanted heart of a patient with dilated cardiomyopathy and a loss-of-function mutation in SCN5A where right sided ST elevation was induced with ajmaline (the patient was not diagnosed with BrS and no ajmaline provocation had been performed in vivo) [49] The disappearance of local activation resulted in monophasic ST elevation in basal RV epicardium, where structural abnormalities (subepicardial adipose and fibrous tissue) were found. Reduction of INa only resulted in the signature Brugada sign following the incorporation of structural discontinuities into a computer model and appeared to be due to excitation failure and activation delay in the subepicardium due to current to load mismatch. This observation leads to an alternative mechanistic explanation for both the classic ECG pattern and arrhythmia, with excitation failure in RV subepicardium causing ST elevation, activation delay at neighbouring sites causing the negative T wave, and conduction block leading to re-entrant arrhythmia.

### Is the Brugada Syndrome a Form of Cardiomyopathy?

The suggestion that BrS may actually be a form of arrhythmogenic right ventricular cardiomyopathy (ARVC), particularly in view of the increasing evidence of structural abnormalities, has been raised. Significant overlap between the two conditions does exist: ajmaline has provoked type 1 changes in ARVC patients [50], and fibrofatty replacement of cardiac myocytes has been reported in patients diagnosed with BrS [51,52]. It is noteworthy, however, that genes associated with ARVC have not been found in Brugada patients. In 1996 Corrado et al investigated members of a single family who possessed the type 1 ECG [53]. Cardiac histopathological examination in the proband revealed myocardial atrophy, transmural fatty replacement, interstitial fibrosis (including fibrosis involving the specialised conducting tissue), although no wall thinning or inflammatory infiltrates associated with classical ARVC were seen. An older sibling had moderate RV dilatation, apical trabecular changes and his right endomyocardial biopsy showed moderate fibrofatty replacement. Some other members of the pedigree had mild to moderate RV and/or RVOT dilatation, wall motion abnormalities and a trabecular pattern on echocardiography. The findings in this family, although not typical of classical ARVC, possess enough pathological features to suggest that there may be a relationship between the two diseases, illustrating the issue of diagnostic classification in the presence of both a typical type 1 ECG and marked structural abnormalities.

## The Role of the Autonomic Nervous System and Other Modulating Factors

A significant proportion of Brugada patients develop ventricular arrhythmias in the resting state or asleep, during which parasympathetic tone predominates. The typical ECG signs are unmasked or exacerbated by vagal stimulation, anti-adrenergic and parasympathomimetic drugs, and attenuated by exercise and isoproterenol infusion [31,54-57]. In some reports, VF was inducible only upon edrophonium (a parasympathomimetic agent) administration, and in other cases, rendered noninducible with isoproterenol [31]. The high frequency component of heart rate variability, representing parasympathetic activity, was observed by Mizumaki et al to be increased in patients exhibiting spontaneous augmentation of ST elevation [56], and by Kasanuki et al to suddenly increase prior to the onset VF in 2 patients [31].

In a study by Babaee Bigi et al, 13 out of 28 patients with a type 1 Brugada ECG pattern were considered to have cardiac autonomic neuropathy, based on 2 non-invasive tests of sympathetic and parasympathetic function [58]. 11 out of these 13 neuropathic patients had a history of cardiac events compared with only 2 of the 15 patients without autonomic dysfunction. Another group identified abnormal regional sympathetic innervation resulting in a relative dominance of parasympathetic activity in the hearts of Brugada patients (the majority with type 1 or type 2 ECGs and arrhythmic events) by examining myocardial adrenergic innervations (utilising 123I-MIBG-SPECT) and presynaptic noradrenaline recycling on PET [59,60].

Although autonomic dysfunction is not a prerequisite of BrS, it appears to be an important modulating factor in baseline ECG and arrhythmia occurrence. Why this is the case has not been elucidated but effects on the activity of ITO and the L-type calcium channel predominant during phase 2 of the action potential have been suggested [59,61]. Effects on phase 2 are particularly noteworthy as together with sodium current, intracellular calcium dynamics play a key role in restitution kinetics, an important factor in the development of VF [62].

Pyrexia has also been seen to unmask the characteristic ECG pattern and there are case reports of fever induced polymoprphic VT in Brugada patients [63-65] and one of ultimately fatal incessant VT in a patient with intractable fever [66]. Dumaine et al found that the Thr 1620 Met missense mutation in SCN5A rendered the sodium channel temperature sensitive, with faster decay and slower recovery from inactivation compared with the wild-type channel in vitro at increasing temperatures [67]. Further mutations rendering the activation and inactivation kinetics of the sodium channel temperature sensitive have been identified since [63-65,68]. Expected to affect sodium current in vivo, it has not been shown how they contribute mechanistically to arrhythmia.

## Conclusions

Both repolarisation and depolarisation abnormalities probably contribute to arrhythmogenesis in the Brugada syndrome. Early repolarisation could cause phase 2 re-entry resulting in closely coupled extrasystoles initiating VT/VF. Conduction delay due to abnormal ion channel kinetics and subtle structural derangements could contribute to this dispersion of repolarisation and provide the substrate for the maintenance of ventricular arrhythmia. These elements may be present to a variable extent in each individual patient, modulated by factors such as vagal tone and pyrexia, and account for both the different degrees of J point elevation on surface ECG. The embryological development of the RVOT and structural changes with age may also lead to intercellular uncoupling allowing repolarisation and depolarisation abnormalities to surface and contribute to individual variation in disease expression.

These issues are also important in the newer J wave disorders responsible for primary VF which are the pathological extension of a normal electrocardiographic variant. Their aetiology probably mirrors that of BrS arising from abnormal ion channel behaviour during phase 0 and phase 1 of the action potential as well as potential dissociation between epi and endocardial repolarisation creating transmural voltage gradients. Indeed BrS may simply reflect a localised RV variant of an extreme form of J point elevation in a spectrum of early repolarisation disorders. These conditions provide an important clinically relevant experimental paradigm which could open the door to new approaches in the treatment and prevention of lethal ventricular arrhythmias.
